# Hybrid Random Forest and Support Vector Machine Modeling for HVAC Fault Detection and Diagnosis

**DOI:** 10.3390/s21248163

**Published:** 2021-12-07

**Authors:** Wunna Tun, Johnny Kwok-Wai Wong, Sai-Ho Ling

**Affiliations:** 1 Faculty of Design, Architecture and Building, University of Technology Sydney, 15 Broadway, Ultimo, NSW 2007, Australia; Wunna.Tun@student.uts.edu.au (W.T.); Johnny.Wong@uts.edu.au (J.K.-W.W.); 2 Faculty of Engineering and IT, University of Technology Sydney, 15 Broadway, Ultimo, NSW 2007, Australia

**Keywords:** heating, ventilation, and air conditioning (HVAC), fault detection and diagnosis (FDD), building management system (BMS), sensors, support vector machine (SVM), random forest (RF)

## Abstract

The malfunctioning of the heating, ventilating, and air conditioning (HVAC) system is considered to be one of the main challenges in modern buildings. Due to the complexity of the building management system (BMS) with operational data input from a large number of sensors used in HVAC system, the faults can be very difficult to detect in the early stage. While numerous fault detection and diagnosis (FDD) methods with the use of statistical modeling and machine learning have revealed prominent results in recent years, early detection remains a challenging task since many current approaches are unfeasible for diagnosing some HVAC faults and have accuracy performance issues. In view of this, this study presents a novel hybrid FDD approach by combining random forest (RF) and support vector machine (SVM) classifiers for the application of FDD for the HVAC system. Experimental results demonstrate that our proposed hybrid random forest–support vector machine (HRF–SVM) outperforms other methods with higher prediction accuracy (98%), despite that the fault symptoms were insignificant. Furthermore, the proposed framework can reduce the significant number of sensors required and work well with the small number of faulty training data samples available in real-world applications.

## 1. Introduction

The heating, ventilation, and air conditioning (HVAC) system in buildings plays an important role in controlling indoor air quality (IAQ) through providing adequate ventilation with filtration and ensuring occupants’ comfort. Typically, the HVAC system is a centralized forced air system, which commonly consists of (i) a central plant with hot water boiler and chiller, (ii) a pump system with hot and chilled water for circulation throughout the circuit of interconnecting pipes, and (iii) an air handling unit (AHU). Operational faults in HVAC systems, including equipment, sensors and control system malfunction, and design problems, often go unnoticed until they end up triggering an equipment-level alarm, leading to a decrease in occupants’ thermal comfort and excessive energy consumption. The study has shown that the HVAC system is the most energy-consuming system in buildings, and almost 30% of the energy consumption in commercial buildings was wasted due to unnoticeable system failures during operation [1]. If faults in HVAC systems can be detected and diagnosed at an early stage, it helps reduce unnecessary waste of electricity. It is, therefore, important to effectively detect and diagnose the defective HVAC equipment for timely maintenance or replacement.

Over the last decades, researchers have been dedicated to exploring various fault detection and diagnosis (FDD) methods using rule-based threshold approaches [2,3], which were typically-found faults. However, the thresholds were difficult to adjust for various HVAC sensors. The adjustment of threshold parameters was both time- and human resource-intense because the threshold must be adjusted over time until the alarm units are effectively disabled. Previously developed fault detection on a physical model, though very useful for complex HVAC systems, was also typically time consuming to retrieve information about equipment and system configuration, building dimensions, and construction materials because of less compatibility and adaptability.

To address the shortcomings of rule-based and physical models, numerous FDD methods with the use of statistical modeling and machine learning have been proposed to detect faults in HVAC systems. For instance, Beghi et al. [4] proposed that a data-driven, semisupervised FDD system should be employed with the use of principal component analysis (PCA), whereas a reconstruction-based approach was used to isolate variables associated with faults. Xiao et al. [5] developed a diagnostic Bayesian network (DBN) FDD method, in which a probabilistic graphical model was used to find the correlation of probabilistic dependencies between faults and evidence. While this method successfully isolated ten common faults, it still required a domain expert in determining the probability of parameters.

With advances in statistical machine learning and information theory, several FDD models have been proposed using artificial neural networks (ANNs) such as adaptive fuzzy neural networks (AFNNs) [6], intelligence swarm-based ANN models [7] with the use of ensemble rapid centroid estimation (ERCE) for automatic feature extraction, and recurrent neural networks (RNN) [8]. In the ANN-based FDD strategy, a two-layer feedforward neural network (NN) trained by an error back-propagation algorithm was used to identify typical AHU sensors and mechanical faults. However, in consideration of expensive computational effort for optimal network structure and parameters, the ANN-based FDD was not a suitable option.

A more cost-effective FDD system that integrates the model-based FDD technique and support vector machine (SVM) was proposed to investigate the three significant faults: the damper fault, cooling coil fault, and supply fan fault. Liang et al. [9] discovered that the SVM-based FDD model has the capacity to isolate these three major faults from normal operation with maximized boundaries between normal and fault classes. To address the shortcomings of current methodologies, Li et al. [10] proposed an SVM-based HVAC fault detection system by learning the consistent nature of different types of faults of HVAC operation. In general, the efficiency and robustness of SVM were very promising when dealing with low-dimensional well-behaved features. Liang et al. considered the SVM-based FDD method to be very promising when dealing with low-dimensional well-behaved features.

To prevent overfitting, an efficient machine learning model, random forest (RF), in combination with extreme gradient boosting (XGBoost) was employed in an FDD system [11]. In the integrated system, the RF was used for feature ranking by its importance, whereas XGBoost was used to classify specific types of faults. Recently, Chakraborty et al. [12] also developed an XGBoost model with a dynamic threshold for the early detection of faults in HVAC systems. Yan et al. [13] proposed an FDD strategy based on classification and regression trees (CART) with an automatic feature extraction strategy. In comparison with other black-box data-driven models, such as ANN and SVM, the decision tree-based classifier is well known for its model interpretability through generating a set of if–then rules in a binary tree form. Despite test results indicating that the proposed CART-based FDD system can effectively recognize relevant failures, some HVAC faults, such as heating coil valve leakage faults and exhaust air dampers stuck in a fully closed position, could not be addressed with the optimized rules.

In order to learn probabilistic relationships between normal and faulty operations, numerous automatic FDD approaches were developed using Hidden Markov Models (HMM) [14] and a DBNs-based FDD model [15,16] for detecting the faults in various HVAC components (e.g., dampers, fans, filters, coils, and sensors). Although these methods consider most typical faults with multiple detectors, they are unfeasible for diagnosing some faults, including exhaust air (EA) dampers stuck in completely open and completely closed positions.

Yan et al. [17] introduced an online fault detection method for chillers by combining extended Kalman filter and recursive one-class SVM (EKF–ROSVM). In this EKF–ROSVM approach, EKF was used to convert every data sample into a vector of parameters to stationarize the dataset, and ROSVM was used to train and detect faults by using parameter vectors. The EKF–ROSVM was further developed from ROSVM and it provided fault detection accuracy improvement over ROSVM.

Recently, Yan et al. [18] proposed a hybrid method by combining the extended Kalman filter with cost-sensitive dissimilar ELM (EKF–CD–ELM) for the fault diagnosis of AHUs. In general, EKF–CD–ELM demonstrated the faster training speed for real-time fault diagnosis with high classification accuracy in the application of fault detection and diagnosis of AHUs. However, EKF–CD–ELM still required the availability of training samples to recognize the faults, and it may be challenging to apply in real-world operations.

To serve as a reference for the development of fault detection technologies for HVAC systems, Lee et al. [19] proposed deep-learning-based fault detection and diagnosis of air-handling units. Rule and convolutional neural networks (RACNN) [20] was introduced for online fault detection and diagnosis of AHUs by combining of rule-based method and CNNs-based method. Though the RACNN approach can automatically and effectively detect the four fault types of laboratory-setup AHU, further investigation was required for real world application.

In fact, an optimized model using SVM and RF-based feature selection has revealed prominent performance and been employed in research in other fields (e.g., medical research) in recent years. For instance, Saifur et al. [21] developed a hybrid SVM and RF-based feature selection optimized model to identify sub-Golgi proteins. In this technique, the most useful features were extracted from protein sequences, and SVM is used to classify the types of protein. Rustam et al. [22] proposed another hybrid approach using SVM and RF classifiers for analysis of gene expression data in chronic kidney disease (CKD). Demidova et al. [23] also proposed the integrated SVM and RF algorithms to improve SVM performance. Given the above research, we employ combined support vector machine (SVM) and random forest (RF) algorithms in detecting fault tasks and failures in HVAC systems. Therefore, the aims of this study are to propose and test a hybrid fault detection and diagnosis for HVAC systems by combining two machine algorithms, random forest and support vector machine.

The novelty of the proposed hybrid random forest–support vector machine (HRF–SVM) approach lies in the method of initial classification; the most significant features are extracted with RF-classifier, then show up at the SVM decision boundary, which uses an intelligent method of binary classification. Our approach is initiated with RF classifier to extract the most important features and fault types classification. With the use of data input from RF with the selected features, the SVM performs as a binary classifier to differentiate between normal and faulty conditions. Then, the decision is fused in an intelligent manner to arrive at a final decision. The proposed HRF–SVM method was validated with the experimental dataset from the ASHRAE RP-1312 project [24], and comparison has been carried out with both traditional intelligent algorithms, including standalone RF, SVM, one vs. rest SVM (OvR-SVM), and recently developed state-of-the-art extended Kalman filter with cost-sensitive dissimilar extreme learning machine (EKF-CS-D-ELM). On a whole, the experimental results demonstrated that the proposed HRF–SVM method significantly improved the performance of fault detection and diagnosis of HVAC system with higher classification accuracy.

The advantages and contributions of proposed fault detection and diagnosis method for HVAC system can be summarized as follows.

1. Based on the historical data obtained from the building management system (BMS), the proposed hybrid method was developed by combining the merits of RF and SVM classifiers. It is not necessary to have prior knowledge of the HVAC system or the cause of the faults, which is advantageous for nonspecialists.
2. In the proposed system, the significant features can be automatically generated with the use of RF classifier, and this enhances the generalization and computational performance. In addition, the proposed method can significantly reduce the number of sensors required for the entire HVAC system to identify the faults efficiently, which is useful in real-world applications.
3. The HVAC system operational data obtained from the BMS is always unbalanced, where the normal conditional data is always more than the faulty conditional data. Since the proposed HRF–SVM uses the merits of both RF and SVM, the imbalanced input data from BMS is effectively handled by implementing RF as a multiclass classifier for fault types classification and SVM as a binary classifier to identify the normal conditions.

In the following section, the theoretical background of SVM and RF classifiers and the proposed HRF–SVM-based FDD is presented. Then, the experiment design and results are discussed, including the comparisons with other FDD methods. Finally, the limitations and suggestions for further research are presented in the concluding remarks.

## 2. Background

### 2.1. Random Forest (RF)

**Figure 1:**
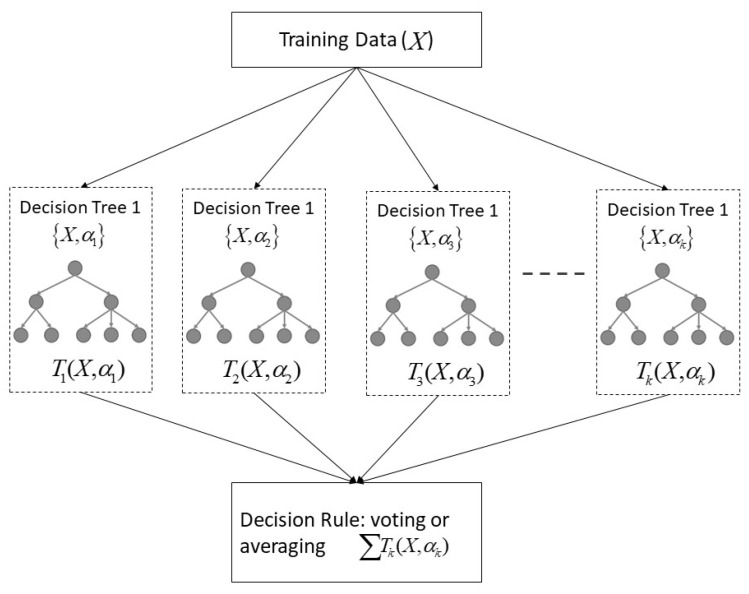
The structure of random forest (RF).

In this section, the random forest (RF) classifier is briefly introduced. The RF classifier, as seen in Figure 1, is a promising ensemble machine learning algorithm which was developed by adding additional layers of decision tree through random sampling of training data and subsets of features while splitting nodes. It has emerged as quite an efficient and robust algorithm that can deal with feature selection, even with higher numbers of features. It was additionally especially proficient while managing missing data, large data without preprocessing, and rescaling. Unlike other black-box algorithms, the RF is trained by a bootstrap aggregating (bagging) algorithm, it improves model stability, accuracy of individual trees, reduces the variance, and overfitting [25]. It is also famous for its model interpretability by generating a set of optimized if–then rules at the end of the training process.

The CART algorithm recursively trains for a given training data set $X=x_{1,}\;x_{2},\ldots ,x_{n}$ with the respond $Y=y_{1,}\;y_{2},\ldots ,y_{n}$ for obtaining a tree predictor $T_{b}$. For each tree $T_{b}\;\left(b\;=\;1,2,\ldots ,B\right)$, it was fitted with a slightly different set of observations, splitting nodes in each tree considering a limited number of features.

1. For b=1 to *B*:
  (a) Draw a bootstrap sample *X* of size *N* from the training data.
  (b) Grow an RF tree $T_{b}$ to the bootstrapped data by recursively repeating the following steps for each terminal node of the tree until the minimum node size $n_{min}$ is reached:
    i. Select *m* variables at random for the *p* variables.
    ii. Pick the best variable/split-point among the *m*.
    iii. Split the node into two daughter nodes.
2. Output the ensemble of the tree ${\left\{T_{b}\right\}}_{1}^{B}$:
  - To make a regression at new points *x*, ${\hat{f}}_{rf}^{B}\left(x\right)=\frac{1}{B}\sum_{b=1}^{B}T_{b}\left(x\right)$.
  - To make a classification ${\hat{C}}_{rf}^{B}\left(x\right)=majorityvote\{{{\hat{C}}_{b}\left(x\right)}_{1}^{B}\}$, whereas ${\hat{C}}_{b}\left(x\right)$ is defined as the class prediction of the *b*th RF tree.

With the use of input dataset *X*, the CART was trained to find a binary partition to increase the response purity in the subspace formed by partition. In RF implementation, it was important to obtain optimal parameters such as the number of trees, the maximum depth of the tree, and the splitting criteria, whereas the Gini impurity was widely used to measure the homogeneity for decision tree [11,26].

### 2.2. Support Vector Machine (SVM)

The SVM is one of the supervised learning algorithms used to solve classification and regression. With the use of the kernel trick technique, it transforms the input data and finds an optimal boundary between positive and negative samples [9,27]. Once the maximized boundary is defined, new samples are then mapped into the same space and predicted to belong to a category based on which side of the gap they fall. In addition, it not only deals with the linear classification problem, but with the use of the kernel trick, it also deals with nonlinear classification. It is able to map low-dimensional inputs to high-dimensional feature spaces through kernel. In particular, the standard soft-margin SVM can be expressed as

(1) $$\min_{\alpha_{i}\gamma_{i}}\frac{1}{2}\sum_{i=1}^{N}\sum_{j=1}^{N}\alpha_{i}\alpha_{j}y_{i}y_{j}K\left(x_{i},x\right)-\sum_{i=1}^{N}\alpha_{i}$$

where $\sum_{i=1}^{N}\alpha_{i}y_{i}=0$ and $0\leq \alpha_{i}\leq C$ for i=1,2,…,L, $\alpha_{i}$ is Lagrange multiplier which was controlled by the penalty factor *C*, $K\left(x_{i},x\right)$ is spectral kernel, which was expressed by linear, polynomial, and Gaussian radial basis function kernels as follows:

(2) $$K\left(x_{i},x\right)=\left\{\begin{array}{c} x_{i}^{T}x\begin{array}{ccc} \begin{array}{cc} \begin{array}{cc} \; & \end{array} & \end{array} & & \end{array}\\ {\left(x_{i}^{T}x+b\right)}^{P},\;\;b>0\\ \exp(-\frac{{∥x_{i}-x∥}^{2}}{2\sigma^{2}}),\;\sigma \neq 0 \end{array}\right.$$

Finally, the output of SVM was calculated by

(3) $$f\left(x\right)=\mathrm{sgn}\left(\sum_{i\notin \vartheta }\alpha_{i}y_{i}K\left(x_{i},x\right)+b\right)$$

where $K\left(x_{i},x\right)$ is a polynomial kernel function that measures the similarity between input pattern $x_{i}$ and training sample *x*, α is the weight parameter, sgn is a signum function, and *b* is the parameter of SVM obtained at the end of the training process.

## 3. Methodology

**Figure 2:**
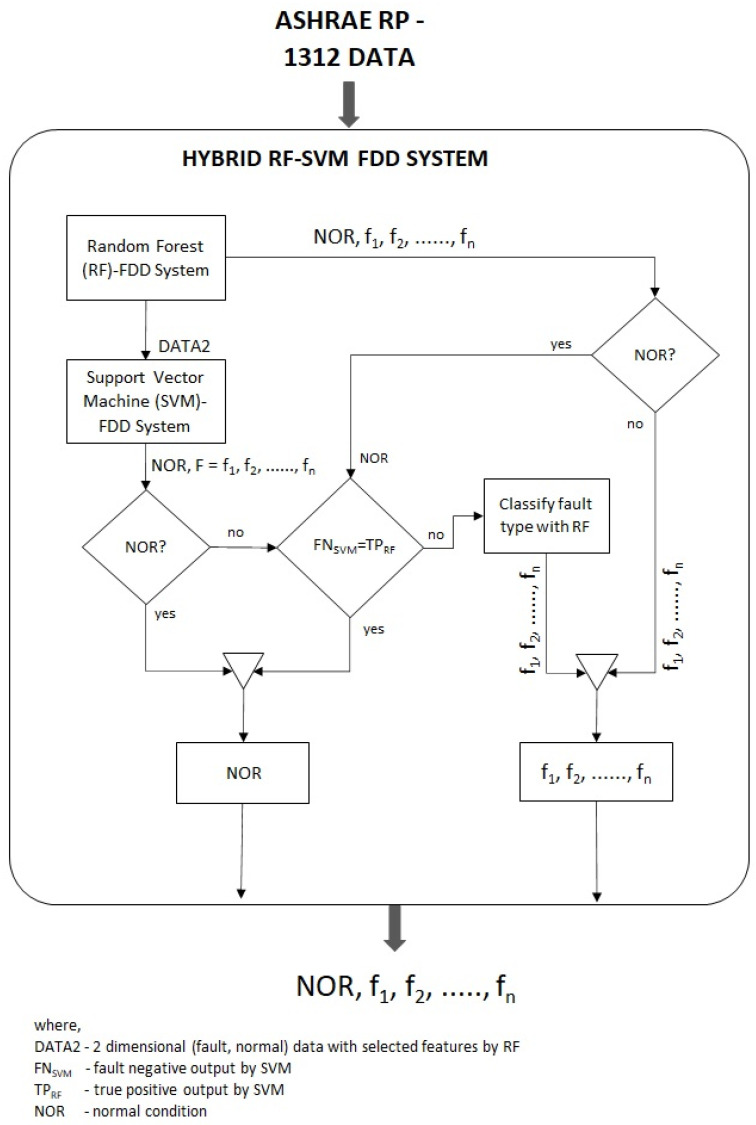
The overview of proposed HRF–SVM FDD system.

In this section, we describe the proposed novel hybrid random forest with SVM (HRF–SVM) method in detail. Practically, SVM is well known for its intelligence classification, especially in handling low-dimensional data, whereas the RF classifier is famous for working with large amounts of missing data and feature selection. The proposed hybrid approach begins with the random forest (RF) classifier for fault detection and diagnosis, followed by the SVM as an auxiliary classifier for improving accuracy. Figure 2 illustrates the framework for fault detection and diagnosis of the HVAC system using an HRF–SVM approach. The sensors reading data, including both faulty and normal operating conditions, is directly input to the proposed system. The proposed HRF–SVM initiates with the RF classifier which classifies different types of faults. The most important features are extracted with the RF classifier which effectively reduces insignificant features and improves the generalization capability of the proposed system. Then, the output data (DATA2) with the selected features from the RF classifier show up at the SVM classifier to maximize the benefits of the algorithm in this hybrid approach. To exploit the merits of SVM, SVM was designed to perform as a binary classifier for normal (NOR) and faulty operation detection in this proposed HRF–SVM, which was achieved with the use of selected RF features, as shown in Figure 2. In (DATA2), the multiple faults, i.e., $f_{1},f_{2},\ldots ,f_{n}$, were explicitly treated as another single fault (F) of the HVAC system, which was denoted as ($F=f_{1},f_{2},\ldots ,f_{n}$) (Figure 2).

As presented in Figure 2, the output of the HRF–SVM $\left(NOR,f_{1},f_{2},\ldots ,f_{n}\right)$ was consolidated by the predicted faults $f_{1},f_{2},\ldots ,f_{n}$ from the first sub-RF system, the predicted normal (NOR) of the second sub-SVM system, and the votes against with the RF (NOR) for the minority fault negative $\left(FN_{SVM}\right)$ samples. In this way, the proposed hybrid system was designed to improve overall performance of the HVAC fault detection and diagnosis system. Differently to other hybrid SVM-based decision trees [21,22,23], the proposed hybrid system was designed to diagnose most common faults of HVAC systems with better accuracy. The most common faults (i.e., 13 different types) for HVAC systems, as described in Table 1, were considered and tested in this study. The effectiveness of the proposed FDD system was compared and analyzed with other machine learning models based on different types of summer fault patterns as reported in the ASHARE RP-1312 project. The detailed experimental results are further elaborated in Section 4.

## 4. Experiment

To test and assess the performance of the proposed HRF–SVM approach, we adopted raw signals, including both faulty and normal data, and selected useful features with an RF classifier. The multivariate analysis is performed using normal vs. faulty data to see the characteristics of the selected features, whereas the HRF–SVM is used to validate the generalization ability and classification accuracy.

### 4.1. Dataset and Parameters

In this experiment, we adopted the dataset from the ASHRAE RP-1312 project [24] to test and validate our proposed hybrid approach. Under different severity levels, both fault-free (normal) and faulty AHU–HVAC experiments were conducted in the Iowa Energy Center Energy Resource Station (ERS). The sensor reading data were collected from the various types of sensor sources, such as temperature sensors, pressure sensors, air flow sensors, humidity sensors, speed sensors, etc., for each component of the HVAC system. The sensor readings were collected at every 1 min for each 24 h of operation. Hence, ~1440 samples were collected during 24 h of operation from each sensor of the HVAC system. The experiment was conducted for 3 normal days, as well as for 13 different fault types on 13 different days.

The experiment setting during summer was divided into three categories: the AHU setting, zone setting, and heating and cooling plants setting. The AHU was scheduled for the period from 6.00 a.m. to 6.00 p.m., whereas the unoccupied period was from 6.00 p.m. to 6.00 a.m. of the following day. The minimum opening for the outdoor air damper was set to 40% open to satisfy the minimum ventilation requirement. It was set to enable the economizer control when the outdoor air temperature (Tsa) was less than 65 ${}^{\circ }$F while the supply air temperature was set to 55 ${}^{\circ }$F. The supply fan speed was controlled to maintain the duct static pressure at 1.4 psi. The return fan was equipped with a speed tracking control device and it was set to maintain 80% of the supply fan speed. The room temperature was set to 70 ${}^{\circ }$F for the occupied period. While the maximum air flow rate was set to 1000 cfm and 400 cfm for exterior and interior zones, the minimum air flow rate was set to 200 cfm for all zones. The testing dates for normal and 13 different fault types are summarized in the third and fourth columns of Table 1.

To validate the proposed HRF–SVM, the data was sampled during the occupied period, which provided 720 data points for 12 h. The sensor readings did not vary substantially outside of the cutoff time period, making it a poor option for modeling and decision-making. As shown in Table 1, a total of 2160 samples were collected for normal data, while fault samples were chosen at random from 40% to 60% of data points, resulting in 300 to 550 samples for each fault. In reality, sensor failures rarely last for long periods of time, and abnormal behaviour can be difficult to detect at times.

### 4.2. Feature Selection

**Figure 3:**
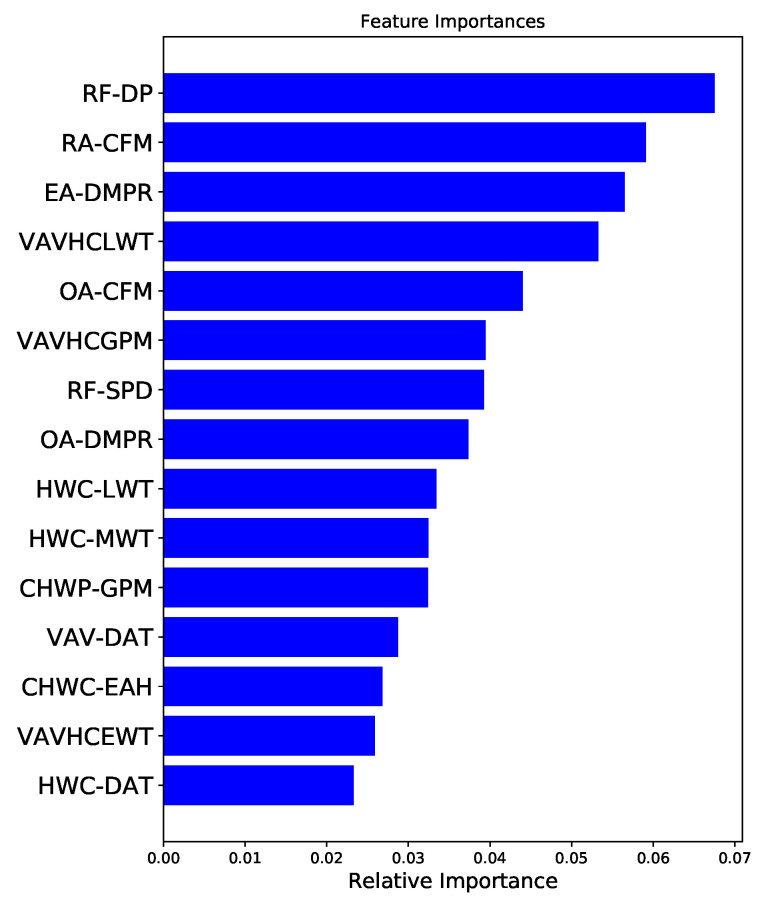
Selected feature importance.

The dataset provided to the model included 160 features which were recorded from the various types of sensors. In our approach, we used RF classifier for the feature selection, and the top 15 most appropriate features were selected, reducing almost 90% of the insignificant features and significantly improving the generalization ability of the proposed hybrid system. The elimination of unnecessary features can also reduce the number of sensors which leads to a cut in the sensors installation cost. As shown in Figure 3, the top selected features were ranked by relative Gini score.

Among all features, the return air fan differential pressure (RF-DP) was found to be the most important feature, followed by return air flow rate (RA-CFM) and exhaust air damper (EA-DMPR) as the second and third most important features, respectively. Apart from the top three most important features, RF also selected the other 12 features, as shown in Figure 3, since they were equally important for the decision-making of the proposed model.

**Figure 4:**
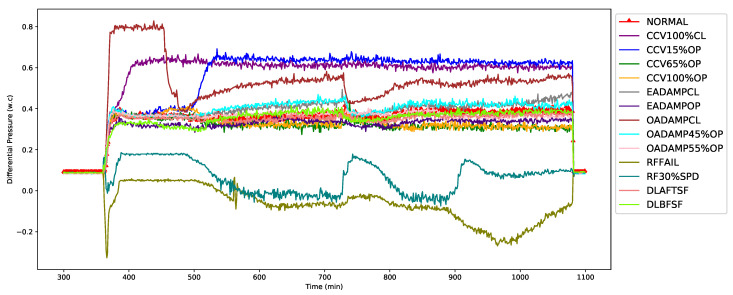
First-top feature: Return air fan differential pressure (RF-DP).

In the AHU system, the return fan was designed to satisfy the 100% static pressure requirements for exhaust air operation, including the return air duct, exhaust air duct, and exhaust air damper. In conjunction with the supply fan, the return fan runs continuously to balance the amount of supplied and exhausted air to the occupied space. As a consequence, the return fan speed is one of the most important factors in AHU operation, and RF-DP was the most important feature selected by the model. As shown in Figure 4, the RF-DP is 0.4 w.c. for the normal operation of the AHU. However, it either increases or decreases when the AHU runs in a faulty condition. Figure 4 also shows that the RF-DP significantly increased to 0.66 w.c. for CCV100%CL and dropped to −0.327 w.c. for the return fan failure (RFFAIL).

**Figure 5:**
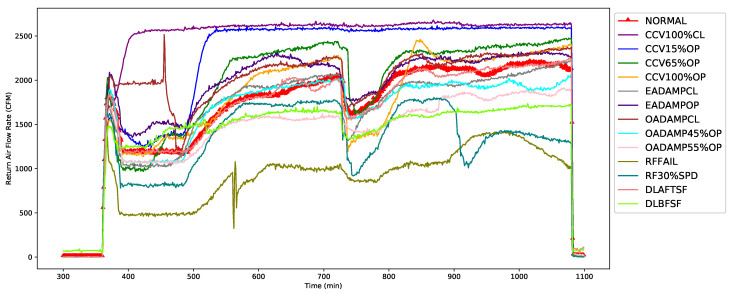
Second-top feature: Return air flow rate (RA-CFM).

As shown in Figure 5, the second-most important feature, RA-CFM, was shown to be directly related to the supply air flow rate, which means that higher the supply air flow required, the higher the return air flow required to maintain the set temperature, humidity, and thermal comfort. Under normal operation, the average RA-CFM was at about 1650 cfm (e.g., at 700 min operation time), whereas it significantly increased to 2590 cfm when the cooling coil valve was in the fully closed fault condition. Similarly, it also changed significantly from 1650 cfm to 920 cfm when the return fan failure occurred.

**Figure 6:**
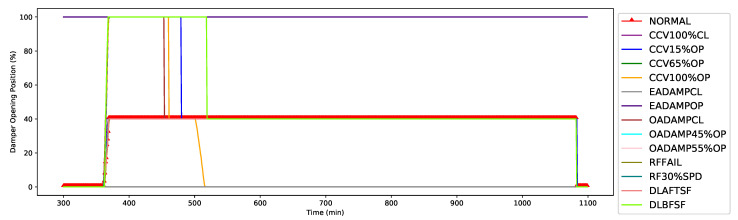
Third-top feature: Exhaust air damper (EA-DMPR).

In addition, the EA-DMPR is the third most significant feature, with relevant Gini score of 0.06, as shown in Figure 3. During the operation of the AHU, the EA-DMPR controls the flow of air volume from the room to the outside environment for optimization of indoor air quality and energy consumption. As illustrated in Figure 6, EA-DMPR was opened 40% during the fault-free (normal) condition. In contrast, it was stuck at fully closed (0% open position) or fully opened (100% open position) for cooling coil faults, outside air damper faults, exhaust air damper faults, or return air fan faults. Likewise, other features, such as VAVHCLWT, OA-CFM, and RA-SPD, changed significantly during the normal vs. faulty conditions.

In the AHU, the VAVHCLWT was largely affected by CCV100%CL and CCV15%OP (cooling coil faults) as supply air temperature failed to maintain at its set point, causing zone air temperature to rise beyond the desired value. Under normal conditions, it was 70 ${}^{\circ }$F, whereas the VAVHCLWT was gradually increased to 82 ${}^{\circ }$F in long run. OA-CFM was maintained at 721 cfm for energy optimization and to improve indoor air quality. However, it became unstable and varied between 0–1050 cfm when running during the faulty conditions. In addition, the faulty conditions highly influenced the RA-SPD, with a maximum speed at 80% and a minimum speed at 30%, compared with a speed of 50–60% under normal conditions. Another important feature, the OA-DMPR opening position, was used to control the amount of fresh air entering the system. In practice, the OA-DMPR opened at 40% for normal AHU operation, while it varied from fully closed to fully opened positions under the faulty operation. Though some of the features were varied only for a few number of faults, consolidation of the top 15 features will give insight to the proposed HRF–SVM FDD system for better understanding as well as decision-making.

### 4.3. Evaluation of Hybrid RF-SVM Model

In this section, the dataset described in Section 4.1 is used to evaluate the feasibility of the proposed HRF–SVM model as presented in Figure 2. Based on the data samples as described in Table 1 (i.e., the fifth column), 80% of the data samples were randomly selected for model training, whereas the remaining 20% were used for testing. Therefore, the proposed model was trained and tested with 5472 and 1368 samples, respectively. Using the training data with 160 features, the HRF–SVM started with the RF classifier that extracted the most significant 15 features, which were used as input for the second-stage SVM classification layer for final classification. On the basis of 13 different types of summer fault patterns, the effectiveness of the proposed HRF–SVM FDD method is compared and evaluated with other FDD models (RF, SVM, one vs. rest-SVM (OvR-SVM), and EKF-CS-D-ELM models).

**Figure 7:**
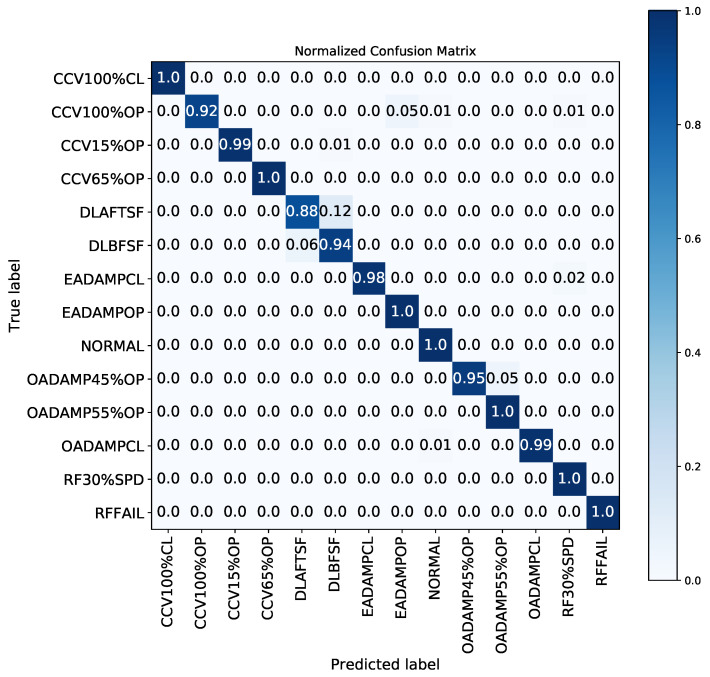
Confusion matrix for proposed HRF–SVM FDD model.

It can be observed that the proposed HRF–SVM is able to diagnose 13 different types of summer faults with higher accuracy (i.e., accuracy of 98%). The confusion matrix in Figure 7 shows a summary of experimental results on the classification of HVAC system faults. It records the diagnosis classification results as well as the number of misclassifications of HVAC system faults with different fault types, severities, and orientations. The confusion matrix in Figure 7 reveals that the HRF–SVM can successfully diagnose faults such as cooling coil valve faults (CCV100%CL, CCV100%OP, CCV15%OP, CCV65%OP) with above 93% accuracy. The proposed hybrid approach can also demonstrate over 98% accuracy in the detection of exhaust air damper faults (EADAMPCL, EADAMPOP), over 95% accuracy for outside air damper faults (OADAMP45%OP, OADAMP55%OP, OADAMPCL), and 100% accuracy in identifying return air fan faults (RFFAIL, RF30%SPD) respectively. As for the detection of duct leakage faults, while the proposed approach can identify 88% and 94% for both DLBFSF and DLAFTSF faults, some DLAFTSF faults were misclassified as DLBSF faults for duct leakage faults.

To further analyze and evaluate the performance of our proposed HRF–SVM method, the precision, recall, and F1-score of the model results are calculated as follows:

(4) $$\mathrm{Precision}=\frac{TP}{\left[TP+FP\right]}\times 100$$

(5) $$\mathrm{Recall}=\frac{TP}{\left[TP+FN\right]}\times 100$$

(6) $$F1\mathrm{-score}=2\times [\frac{\mathrm{Precision}\times \mathrm{Recall}}{\mathrm{Precision}+\mathrm{Recall}}]\times 100$$

where precision is the ratio of correctly predicted positive instances to total predicted positive instances, recall is the ratio of correctly predicted positive instances to all actual positive instances, F1-score is the weighted average of precision and recall, TP is the number of true positive instances, FP is the number of false positive instances, and FN is the number of false negative instances.

Table 2 describes the detail of precision rates, recall rates, and F1-score rates for the experimental results of the proposed HRF–SVM method. As shown in Table 2, except for the precision rate of DLBFSF being 87%, the precision rates for others are between 93–100%. The proposed HRF–SVM also achieves recall rates above 95%, apart from DLAFTSF, CCV100%OP, and DLBFSF, for 88%, 93%, and 94%. Therefore, the experimental results illustrate that the performance of our proposed HRF–SVM is excellent for fault detection and diagnosis of HVAC systems. In addition, F1-score rates are calculated to determine the model performance for the handling of unbalanced data. Generally, a high F1-score means that the model performs well in the handling of unbalanced data. The detailed F1-score results in Table 2 demonstrate that our proposed HRF–SVM performs well in the handling of unbalanced input dataset.

### 4.4. Comparison of Performance of HRF–SVM Model with Other Methods

For the RF classifier, the experiment started with an RF classifier using a raw sensor signal with 160 features to compare the performance of the models. The experimental result, as shown in Table 3, revealed that the RF performed well for detecting and diagnosing HVAC faults, with an accuracy of 86%. It also provides promising results for cooling coil valve faults (CCV15%OP, CCV65%OP, CCV100%CL), return air fan faults (RFFAIL and RF30%SPD), outdoor air damper faults (OADAMP45%OP, OADAMP55%OP, and OADAMPCL), exhaust air damper faults (EADAMPCL, EADAMPOP), and duct leakage before supply air fan (DLBFSF). However, the RF classifier performed considerably lower than the proposed HRF–SVM approach in classifying the duct leakage after supply fan (DLAFTSF) fault and normal conditions.

For the performance of the SVM-based model, the model was trained and evaluated with the input dataset to compare the performance with other approaches. As shown in Table 3, the SVM-based FDD system achieves better results by isolating the faults from normal conditions by obtaining 100% accuracy. However, the SVM approach failed to detect when outside air damper partially opened to 45% and 55% faults (OADAMP45%OP, OADAMP55%OP). In addition, the SVM approach provided significantly low accuracy in classifying some of the fault types, including DLAFTSF (4% accuracy) (duct leakage after supply fan), DLBFSF (7% accuracy) (duct leakage before supply fan), and CCV100%OP (65% accuracy) (cooling coil valve 100% opened) faults. The results show that, while the SVM performed well in classification for normal conditions, it only has an accuracy of 77% in overall HVAC system fault detection.

For the performance of the one vs. rest SVM (OvR-SVM) model, the experiment results showed that the OvR-SVM model performed well for some cases by giving model accuracy of 81%. In spite of 81% model accuracy, the OvR-SVM approach only achieved relatively low accuracy in detecting some fault types, including outside air damper partially opened faults (4% for OADAMP45%OP, 13% for OADAMP55%OP), exhaust air damper fully closed faults (25% for EADAMPCL), and duct leakage faults (26% for DLAFTSF, 54% for DLBFSF).

On top of the experiments, the proposed HRF–SVM also compared with the recently developed hybrid method combining the extended Kalman filter with cost-sensitive dissimilar ELM (EKF-CS-D-ELM) [18], which used the same dataset as our proposed model. Overall, the experiment results shown in Table 3 illustrate that our proposed method achieved better performance in diagnosing different types of faults than the recently developed EKF-CS-D-ELM method.

## 5. Conclusions

This paper presented a novel hybrid fault detection and diagnosis approach for HVAC systems, which consists of traditional random forest (RF) and SVM classifiers. Traditionally, the SVM has its own advantages when dealing with low-dimensional features, and the RF can automatically perform feature selection, thereby saving time and human effort. In our model, the useful features were firstly extracted by an RF classifier, followed by the adoption of the SVM to isolate normal operations as well as 13 different types of faults into cooling coil valve fault, exhaust air damper fault, outside air damper fault, duct leakage, and return air fan failure. Fault detection and diagnosis performance of our proposed hybrid approach and various other current approaches, including the RF, SVM, OvR-SVM, and EKF-CS-D-ELM, were compared and analyzed. Among the top 15 RF-ranked features, this study showed that the return air fan differential pressure (RF-DP), return air flow rate (RA-CFM), and exhaust air damper (EA-DMPR) were found to be the most significant features, while the rest were inclusively important. The experimental results showed that the proposed hybrid RF-SVM approach in this study successfully diagnosed 13 different types of HVAC faults by achieving higher accuracy at 98%.

Though our method achieves promising results, it still has some limitations. The proposed hybrid system takes longer processing time than the standalone classifier models. The proposed model was only tested for the summer season. In future research, we aim to yield further improvements with a shorter execution time and improved efficiency. Moreover, we strive to investigate the performance of the proposed method in all different seasons and to also test the proposed hybrid model on alternative HVAC systems.

## Figures and Tables

**Table 1:** Summary of AHU faults considered in the proposed FDD model.

Fault	Abbreviation	Description	Test Date	Sample
			19 August 2007	
F0	NORMAL	Normal condition	25 August 2007	2160
			04 September 2007	
F1	CCV15%OP	Cooling coil valve 15% opened	01 September 2007	432
F2	CCV65%OP	Cooling coil valve 65% opened	02 September 2007	432
F3	CCV100%CL	Cooling coil valve fully closed	27 August 2007	576
F4	CCV100%OP	Control coil valve fully opened	31 August 2007	360
F5	DLAFTSF	Duct leak after supply air fan	07 September 2007	360
F6	DLBFSF	Duct leak before supply air fan	08 September 2007	360
F7	EADAMPCL	Exhaust air damper closed	20 August 2007	288
F8	EADAMPOP	Exhaust air damper opened	21 August 2007	288
F9	OADAMP45%OP	Outside air damper 45% opened	05 September 2007	288
F10	OADAMP55%OP	Outside air damper 55% opened	06 September 2007	288
F11	OADAMPCL	Outside air damper closed	26 August 2007	360
F12	RFFAIL	Return air fan failure	23 August 2007	360
F13	RF30%SPD	Return air fan 30% fixed speed	22 August 2007	288

**Table 2:** Classification report for proposed HRF–SVM.

Fault	Precision (%)	Recall (%)	F1-Score (%)
NORMAL	100	100	100
CCV15%OP	100	99	99
CCV65%OP	100	100	100
CCV100%CL	100	100	100
CCV100%OP	100	93	96
DLAFTSF	94	88	91
DLBFSF	87	94	90
EADAMPCL	100	98	99
EADAMPOP	93	100	96
OADAMP45%OP	100	95	97
OADAMP55%OP	95	100	97
OADAMPCL	100	99	99
RFFAIL	100	100	100
RF30%SPD	97	100	98

**Table 3:** Comparison results for fault detection and diagnosis of HVAC systems.

Fault	HRF–SVM (%)	RF (%)	SVM (%)	OvR-SVM (%)	EKF-CS-D-ELM (%)
NORMAL	100	52	100	98	NA
CCV15%OP	99	100	81	99	96.7
CCV65%OP	100	100	92	91	99.1
CCV100%CL	100	100	98	100	96.4
CCV100%OP	93	88	65	82	95.9
DLAFTSF	88	76	4	26	94.4
DLBFSF	94	93	7	54	97.6
EADAMPCL	98	100	78	25	95.2
EADAMPOP	100	98	98	100	91.3
OADAMP45%OP	95	89	0	4	NA
OADAMP55%OP	100	98	0	13	92.4
OADAMPCL	99	100	91	99	91.3
RFFAIL	100	100	100	100	93.1
RF30%SPD	100	100	100	100	84.1
Model Accuracy	98	82	77	81	94

## Data Availability

Data was obtained from ASHRAE 1312-RP project and are available from https://www.techstreet.com/ashrae/standards/rp-1312-tools-for-evaluating-fault-detection-and-diagnostic-methods-for-air-handling-units?gatewaycode=ashrae&productid=1833299 (accessed on 1 December 2021) with the permission of ASHRAE.
